# Incorporation of EPA and DHA into plasma phospholipids in response to different omega-3 fatty acid formulations - a comparative bioavailability study of fish oil vs. krill oil

**DOI:** 10.1186/1476-511X-10-145

**Published:** 2011-08-22

**Authors:** Jan Philipp Schuchardt, Inga Schneider, Henrike Meyer, Juliane Neubronner, Clemens von Schacky, Andreas Hahn

**Affiliations:** 1 Institute of Food Science and Human Nutrition, Leibniz University Hannover, Germany; 2 Preventive Cardiology, Medizinische Klinik und Poliklinik Innenstadt, Ludwig Maximilians University Munich, Germany

**Keywords:** bioavailability, absorption, uptake, ethyl esters, re-esterified triacylglycerides, fish oil, krill oil

## Abstract

**Background:**

Bioavailability of omega-3 fatty acids (FA) depends on their chemical form. Superior bioavailability has been suggested for phospholipid (PL) bound omega-3 FA in krill oil, but identical doses of different chemical forms have not been compared.

**Methods:**

In a double-blinded crossover trial, we compared the uptake of three EPA+DHA formulations derived from fish oil (re-esterified triacylglycerides [rTAG], ethyl-esters [EE]) and krill oil (mainly PL). Changes of the FA compositions in plasma PL were used as a proxy for bioavailability. Twelve healthy young men (mean age 31 y) were randomized to 1680 mg EPA+DHA given either as rTAG, EE or krill oil. FA levels in plasma PL were analyzed pre-dose and 2, 4, 6, 8, 24, 48, and 72 h after capsule ingestion. Additionally, the proportion of free EPA and DHA in the applied supplements was analyzed.

**Results:**

The highest incorporation of EPA+DHA into plasma PL was provoked by krill oil (mean AUC_0-72 h_: 80.03 ± 34.71%*h), followed by fish oil rTAG (mean AUC_0-72 h_: 59.78 ± 36.75%*h) and EE (mean AUC_0-72 h_: 47.53 ± 38.42%*h). Due to high standard deviation values, there were no significant differences for DHA and the sum of EPA+DHA levels between the three treatments. However, a trend (*p = *0.057) was observed for the differences in EPA bioavailability. Statistical pair-wise group comparison's revealed a trend (*p *= 0.086) between rTAG and krill oil. FA analysis of the supplements showed that the krill oil sample contained 22% of the total EPA amount as free EPA and 21% of the total DHA amount as free DHA, while the two fish oil samples did not contain any free FA.

**Conclusion:**

Further studies with a larger sample size carried out over a longer period are needed to substantiate our findings and to determine differences in EPA+DHA bioavailability between three common chemical forms of LC n-3 FA (rTAG, EE and krill oil). The unexpected high content of free EPA and DHA in krill oil, which might have a significant influence on the availability of EPA+DHA from krill oil, should be investigated in more depth and taken into consideration in future trials.

## Background

Scientific and regulatory bodies recommend an increased intake of EPA+DHA for cardiovascular prevention or during pregnancy [1-3]. Because a number of large intervention trials were conducted using 1 g EPA+DHA ethyl-ester (EE)/d, the American Heart Association recommends 1 g EPA+DHA/d in fish or as capsules [4]. As a result of a consensus conference, increasing DHA intake by 200 mg/d during pregnancy has been recommended [2]. However, in natural fish oil, EPA+DHA are bound in triacylglycerides (TAG) whereas many fish oil capsules contain the fatty acids (FA) bound in EE or re-esterified TAG (rTAG). Implicitly, the recommendations assume that different chemical forms of EPA and DHA have comparable bioavailability.

Recently, we demonstrated that a six-month supplementation of identical doses of EPA+DHA (1.67 g/d) leads to a faster and higher increase of the omega-3 index - the percentage of EPA and DHA in red cell membranes reflecting the long-term intake and n-3 FA status of an individual [5] - when consumed as rTAG than when consumed as EE [6]. After six months, the omega-3 index increased significantly higher in the rTAG-group than in the EE-group [197% vs. 171% [p < 0.01]). Our results were in line with data from a short-term study of two weeks duration measuring the FA compositions of serum cholesterol-esters, TAG and phospholipids (PL): relative bioavailability of EPA+DHA from rTAG was superior (124%) compared with cod liver oil (100%), whereas the bioavailability from EE was inferior (73%) [7]. Bioavailability of free FA and natural fish oil TAG was similar to cod liver oil [7]. Smaller trials carried out earlier had inconsistent results [8-13]. A direct comparison of krill oil to fish oil TAG noted similar changes in plasma FA, while fish oil reduced the blood pressure and krill oil did not [14]. In another comparison, the dose of EPA+DHA in krill oil was 62.8% of that in fish oil, but similar changes in plasma EPA+DHA and of a lipid panel were observed [15].

To the best of our knowledge, no study has attempted to compare the bioavailability of identical doses of EPA+DHA from krill oil (largely PL) to that of other chemical forms. Therefore, in the present study, we compared single doses of three commonly available chemical forms of EPA and DHA, rTAG, EE and PL, in a double-blinded crossover design. We used changes of the FA compositions in plasma PL within 72 h following the ingestion of a single dose of EPA+DHA as a proxy for bioavailability. The crossover design was considered as particularly suitable to assess the comparative bioavailability of EPA + DHA from n-3 FA formulations, since inter-individual variability, which is frequently observed after the administration of n-3 FA supplements, can be minimized. Additionally, we analyzed the proportion of free EPA and DHA in the used supplements, since free long-chain (LC) FA are known to possess a greater bioavailability compared to other n-3 FA formulations.

## Methods

### Subjects and study design

Healthy young men were recruited from the general population by an advertisement. The inclusion criteria for participating in the study were male gender, an age between 20 and 50 years and a body mass index (BMI) between 20 and 28 kg/m^2^. Diagnosis or suspicion of gastrointestinal disorders, high intake of oily fish (> 2 times per week), intake of dietary supplements (e.g. n-3 FA, phytosterols, polyglucosamin) and intake of lipid-lowering drugs two weeks before and during intervention were defined as exclusion criteria. Inclusion and exclusion criteria were assessed via structured questionnaires before the intervention period and on the intervention days. Subjects were instructed to avoid foods rich in n-3 FA to minimize intake variability. Restricted foods included fish, seafood, and vegetable oil.

Twelve male volunteers (age 31 ± 5 years) were given one of three highly purified EPA and DHA rich oils in a crossover design, each intervention day separated by 14 days wash-out period. The allocation of subjects to different treatments was carried out in a randomized order to avoid treatment order effects. The three different supplements were: fish oil capsules consisting of EPA+DHA as rTAG, fish oil capsules consisting of EPA+DHA as EE and krill oil capsules consisting of EPA+DHA mainly as PL. The two fish oil supplements (rTAG and EE) were provided by Dr. Loges + Co. GmbH, Winsen, Germany, while the krill oil was a commercially available supplement (NKO^®^) from Neptune Technologies & Bioressources Inc., Québec, Canada.

During every intervention day, each subject ingested the allocated capsules at 7:00 a.m. after an overnight fast. Capsules were given with a standardized breakfast that contained 30.1 g of fat, 29.6 g of protein, 67.9 g of carbohydrate, and 2.7 MJ of energy. The total fat intake (breakfast + capsules) was 33.5 g for the rTAG and the EE treatment and 37.1 g for the krill oil treatment. During the intervention day, subjects consumed standardized meals over 24 h, which were personally adjusted according to weight. Meals were consumed 3, 5, 7, 10, 12, and 24 hours after capsule ingestion. Blood samples were taken initially (predose at baseline) and 2, 4, 6, 8, 24, 48, and 72 h after the intake of the capsules. Volunteers were only allowed to drink water during the intervention day.

This investigator initiated study was designed and conducted in accordance with the principles of the GCP Guidelines laid down in the Declaration of Helsinki, and was approved by Freiburger Ethik-Komission International, Germany. Written informed consent was obtained from all subjects. The trial was registered at ClinicalTrials.gov (ID: NCT01214278).

### Composition of study supplements and total EPA+DHA intake

The composition and amounts of the supplements are given in Table 1. While the total EPA+DHA intake was 1680 mg for all three treatments, there were marginal differences in the individual intake of EPA and DHA between the three treatments. The total EPA intake was 1080 mg for the rTAG and EE treatment and 1050 mg for the krill oil treatment, while the total DHA intake was 672 mg for the rTAG and EE treatment and 630 mg for the krill oil treatment.

**Table 1:** Dose and composition of capsules administered in the three study supplements

	rTAG		EE		Krill oil	
	per capsule	per day (4 capsules)	per capsule	per day (4 capsules)	per capsule	per day (14 capsules)
EPA	252 mg	**1008 mg**	252 mg	**1008 mg**	75 mg	**1050 mg**
DHA	168 mg	**672 mg**	168 mg	**672 mg**	45 mg	**630 mg**
EPA+DHA	420 mg	**1680 mg**	420 mg	**1680 mg**	120 mg	**1680 mg**
total n-3 FA	504 mg	**2016 mg**	504 mg	**2016 mg**	150 mg	**2100 mg**
total fat	850 mg	**3400 mg**	850 mg	**3400 mg**	500 mg	**7000 mg**

Abbreviations: rTAG: re-esterified triacylglycerides; EE: ethyl esters

### Quantitative analysis of free FA in fish and krill oil samples

Analysis of free FA (FFA) were performed in an external laboratory (Lipidomix GmbH, Berlin, Germany). 10 mg oil of each product sample was dissolved in 1 mL ethanol. FA were measured through reversed phase HPLC-Mass spectrometry with electrospray ionization in negative mode. The Agilent 1200SL/6460 system, operated in MS2Scan mode, was equipped with a Zorbax SB-C18 30 × 2.1 mm, 1.8 μm column, while Methanol/10 mM ammonium acetate in water was used as solvent. FA were identified by comparison with a FA standard mixture. The calibrated range was 1-1000 mg of each FA.

### Anthropometric measurements and blood sampling

Subjects were examined by trained professionals according to standardized methods at the Institute of Food Science and Human Nutrition of Leibniz University Hannover, Germany. Body weight was measured using a calibrated scale (SECA, Hamburg, Germany), body height was surveyed via tape measure and BMI was calculated as weight (kg)/height (m^2^). Body weight and height were measured each intervention day. The blood samples were drawn using EDTA-monovettes (Sarstedt, Germany).

### Plasma phospholipid fatty acid analyses in blood samples

For plasma preparation, blood samples were directly centrifuged (2000 g, 10 min, 10°C), and plasma taken off. Prepared samples were stored at -70°C and shipped on ice to the laboratory (Omegametrix GmbH, München, Germany) after completion of the study. Plasma PL FA composition (primary endpoint) was analyzed as described previously [16] with minor modifications. Lipids were extracted, and PLs purified by use of a Sep-Pak C-18 mini cartridge (Waters, Eschborn, Germany). FA methyl esters were formed by acid hydrolysis, and were analyzed by capillary gas-liquid chromatography on a GC2010 Gas Chromatograph (Shimadzu, Duisburg, Germany) equipped with a SP2560, 100-m column (Supelco, Bellefonte, PA) using hydrogen as carrier gas. FA were identified by comparison with a FA standard mixture. Results are expressed as % of total FA in plasma PL.

### Mathematical and statistical analyses

Data are presented as mean ± standard deviation for continuous variables, and number of subjects (n) and percentage (%), respectively for categorical variables. All plasma levels were corrected to baseline levels. Areas under the curve (AUC) of EPA+DHA were calculated for each person during each intervention period according to the trapezoidal rule. Time (t_max_) to maximum serum concentrations (c_max_) were identified by visual inspection of the data. Data was found to be normally distributed using the Kolmogorov-Smirnov test, and therefore parametric tests were used for the statistical analyses. AUC, c_max _and t_max _were compared by ANOVA with post hoc testing according to Bonferroni's method. Values of *p *≤ 0.05 were considered to be statistically significant, while a trend was defined as *p *≤ 0.1. Statistical analyses were carried out with SPSS software (version 18.0; SPSS Inc, Chicago, IL, USA).

## Results

### Subject characteristics

Twelve healthy males participated in this study. Their mean age was 31 ± 5 years, mean height was 1.84 ± 0.09 m, mean weight was 83.3 ± 9.4 kg, and mean BMI was 24.6 ± 2.2 kg/m^2^. Due to the crossover design baseline levels were not significantly different between the treatments.

### EPA and DHA in plasma phospholipids

The amounts of EPA and DHA, as well as total n-3 FA, in plasma PL (mean ± standard deviation) at baseline and 24 h after ingestion of the different marine n-3 FA formulations are given in Table 2. The n-3 FA concentrations in plasma PL were at their highest after 24 h. Differences in plasma PL levels between rTAG, EE and krill oil are demonstrated in Table 3, which shows the EPA, DHA and total n-3 FA gradients as AUC_0-72_, c_max _and t_max _values.

**Table 2:** Plasma phospholipid EPA, DHA, and total n-3 FA levels (mean *± *standard deviation, % of total FAs) at baseline and 24 h after ingestion of supplements

	Baseline (%)			After 24 h (%)		
	
Fatty acids	rTAG	EE	Krill oil	rTAG	EE	Krill oil
EPA	0.92 ± 0.34	0.94 ± 0.30	0.79 ± 0.25	1.68 ± 0.30	1.82 ± 0.38	1.80 ± 0.36
DHA	2.61 ± 0.23	2.71 ± 0.64	2.56 ± 0.60	3.00 ± 0.52	2.90 ± 0.63	3.03 ± 0.48

EPA+DHA	3.53 ± 0.93	3.66 ± 0.77	3.35 ± 0.80	4.68 ± 0.52	4.72 ± 0.65	4.83 ± 0.59

total n-3 FA	4.70 ± 1.14	4.82 ± 0.98	4.38 ± 0.87	5.89 ± 0.55	5.93 ± 0.63	5.98 ± 0.63

Abbreviations: rTAG: re-esterified triacylglycerides; EE: ethyl esters

**Table 3:** Plasma phospholipid EPA, DHA, and total n-3 FA levels (mean *± *standard deviation, AUC [%*h], c_max _[%] and t_max _[t]) 72 h after ingestion of supplements

		rTAG	EE	Krill oil	p *
	EPA	37.05 ± 15.97	38.68 ± 18.60	53.62 ± 17.31	0.057
	
AUC [%*h]	DHA	22.73 ± 26.23	8.85 ± 22.57	26.41 ± 29.54	0.246
	
	EPA+DHA	59.78 ± 36.75	47.53 ± 38.42	80.03 ± 34.71	0.119
	
	total n-3 FA	66.35 ± 37.53	49.49 ± 46.88	88.40 ± 42.81	0.134

	EPA	0.98 ± 0.26	0.79 ± 0.51	1.18 ± 0.45	0.095
	
c_max _[%]	DHA	0.42 ± 0.48	0.21 ± 0.18	0.40 ± 0.41	0.323
	
	EPA+DHA	1.12 ± 0.66	0.72 ± 0.76	1.51 ± 0.69	0.039
	
	total n-3 FA	1.44 ± 1.06	0.73 ± 0.76	1.44 ± 0.89	0.110

	EPA	11.5 ± 7.6	10.2 ± 8.6	7.8 ± 5.8	0.500
	
t_max _[h]	DHA	5.0 ± 6.2	9.8 ± 20.6	4.2 ± 6.6	0.538
	
	EPA+DHA	10.5 ± 10.2	9.0 ± 9.2	7.1 ± 6.0	0.649
	
	total n-3 FA	16.2 ± 20.2	9.0 ± 9.2	8.0 ± 8.2	0.307

Abbreviations: rTAG: re-esterified triacylglycerides; EE: ethyl esters; AUC: area under the curve

* ANOVA

The comparison of AUC_0-72 _values shows that the EPA, DHA, EPA+DHA and total n-3 FA levels in plasma PL were higher after the krill oil treatment compared to rTAG and EE. Furthermore the DHA, EPA+DHA and total n-3 FA uptake from the rTAG formulation was higher compared to EE, while the EPA uptake was higher after EE treatment than after rTAG treatment. However, due to high standard deviation values, the observed differences were not significant. A trend (*p *= 0.057) was observed for EPA. Post-hoc tests revealed a trend (*p *= 0.086) between rTAG and krill oil.

The c_max _levels for EPA+DHA differed significantly (*p *= 0.039), although statistical pair-wise group comparison showed only significant differences between krill oil and EE (*p *= 0.034). No significant differences in c_max _levels were observed for EPA, DHA and total n-3 FA between the treatments. However, a trend (*p *= 0.095) in c_max _levels was observed for EPA. Post-hoc tests revealed a trend (*p *= 0.094) between krill oil and EE. No significant differences in t_max _levels were observed between the treatments (rTAG, EE and krill oil).

**Figure 1:**
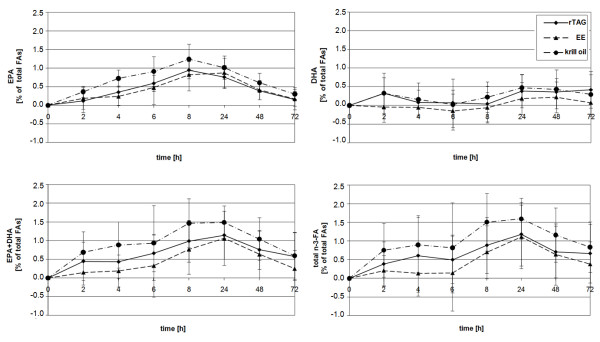
**Increase of n-3 FA concentrations in plasma PL after the ingestion of supplements**. Healthy young males received n-3 FA as fish oil rTAG (solid line), EE (dash line), or krill oil (dash dot line) supplements in a crossover manner. Results show change of EPA, DHA, EPA+DHA, and total n-3 FA levels in plasma PL (% of total FAs, mean ± standard deviation) from baseline concentrations over 72 h.

Figure 1 illustrates the time-dependent courses of the mean percent change of EPA, DHA, EPA+DHA, and total n-3 FA levels in plasma PL from baseline to 72 h post-consumption. Each n-3 FA formulation produced an increase in EPA, DHA and total n-3 FA content in plasma PL, except for DHA after EE treatment. In the latter treatment the DHA values dropped a priori and reached a positive range approximately 12 h after ingestion of the supplement.

### Proportion of free FA in fish and krill oil samples

While no FFA were determined in the two fish oil samples rTAG and EE, the krill oil sample contained 33 mg EPA/g (22% of the total EPA amount) and 19 mg DHA/g (21% of the total DHA amount).

## Discussion

In a randomized crossover study in healthy volunteers, we observed the highest incorporation of EPA, DHA and total n-3 FA into plasma PL after the ingestion of krill oil (PL and FFA), followed by rTAG derived from fish oil, again followed by EE. Due to high standard deviation values, the differences were not significant. However, a trend was observed for EPA.

Few studies investigated differences in EPA+DHA bioavailability between krill oil and fish oil TAG. One randomized, double-blind, placebo-controlled trial over 4 weeks compared differences in EPA+DHA uptake between krill oil and fish oil TAG by analyzing total plasma FA composition [14]. While subjects on krill oil received 216 mg/d EPA and 90 mg/d DHA, subjects on menhaden oil received 212 mg/d EPA and 178 mg/d DHA. Total plasma EPA and DHA levels in both n-3 FA groups increased similarly. Consistent with our results, the EPA uptake from krill oil was higher compared to menhaden oil. On the other hand, the DHA uptake from menhaden oil was greater than in the krill oil group, probably due to the higher dose given. In contrast to the present study, doses of n-3 FA were not well matched, obviating strong conclusions about bioavailability. Of note, systolic blood pressure declined only in the menhaden oil group, possibly reflecting the fact that DHA, but not EPA, lowers blood pressure [17].

More recently, Ulven et al. [15], compared the uptake of n-3 LC PUFA in the form of TAG from fish oil to krill oil in a randomized, parallel group study over 7 weeks, and used total plasma FA composition as an endpoint. Although the EPA+DHA doses used were different (543 mg in krill oil, 864 mg in fish oil), changes in total plasma FA and in a plasma lipid panel were similar. Although, again, differences in dose obviate clear conclusions on bioavailability, the data indicates that absorption of EPA+DHA from krill oil was superior to fish oil TAG, which would be in keeping with our results.

While the studies from Maki et al. [14] and Ulven et al. [15] analyzed total plasma FA composition, we used changes in plasma PL FA composition after a single dose treatment as a proxy for bioavailability. There is some disagreement as to what parameter reflects the best bioavailability of LC n-3 FA. In terms of LC n-3 FA, red blood cells reflect tissue composition, at least of the heart, but probably also of other organs [18]. It remains to be demonstrated whether our findings can be reproduced in a longer study focusing on red blood cell FA composition, e.g. by using the omega-3 index.

The mechanisms underlying the larger incorporation of EPA+DHA from krill oil into plasma PL remain unknown and might be due to several reasons. First of all, it was rather surprising that the used krill oil contained a remarkable amount of EPA (22% of total EPA amount) and DHA (21% of total DHA amount) as FFA. It is assumed that the rest of EPA (78%) and DHA (79%) in the krill oil sample is bound to phospholipids, while only negligible contents are bound in TAGs. So far it was generally accepted that EPA and DHA from krill oil is predominantly bound in PL. The unexpected high content of FFA in krill oil might improve the bioavailability of EPA+DHA from krill oil. A greater EPA or DHA bioavailability of FFA compared to rTAG or EE, respectively, has been shown in earlier studies [8,9,19], whereas Dyerberg et al. [7] found the EPA+DHA bioavailability from FFA to be equivalent to natural TAGs. Likewise, a recent study demonstrated a markedly enhanced bioavailability of n-3 FFA over EE in overweight patients on a low-fat diet, which is recommended for patients with hypertriglyceridemia [20].

On the other hand, the major proportion of EPA+DHA in krill oil is bound in PL, and the intestinal absorption of PL bound FA might be more efficient compared to rTAG and EE. The digestion of FA esterified as PL is carried out mainly by pancreatic phospholipase A2 (pPLA2) and other pancreatic lipases. pPLA2 interacts with PL at the sn-2 position yielding FFA and lyso-phosphatidylcholine, which are absorbed by the enterocytes as parts of mixed micelles [21]. PL exhibit an amphiphilic character and therefore emulsification properties. As a result, PL influence the surface composition of fat droplets, which possibly facilitates the binding of hydrolyzing enzymes and hence the digestion [22]. Similarly, their presence is essential for the formation of mixed micelles. It is possible that higher contents of PL support this formation process, leading to an enhanced absorption of lipids. The activities of other PL hydrolyzing lipases are likely to play a meaningful role in the digestion of PL. Studies with pPLA2-KO mice indicates that pPLA2 deficiency does not affect PL hydrolysis and absorption in contrast to TAG [23], possibly because its activity is compensated by other PL hydrolyzing enzymes [24]. Thus the hydrolyzing capacity of these lipases might also contribute to an efficient digestion of dietary LC n-3 FA PL.

After absorption into enterocytes, the metabolism of LC PUFA involves re-esterification into TAG (2-monoglyceride pathway) and PL (α-glycerophosphate pathway), as well as the formation of chylomicrons for further transport [25,26]. It can only be speculated if the higher EPA+DHA plasma PL responses after krill oil ingestion is a result of an intensified incorporation of EPA+DHA into PL in consequence of an increased presence of lyso-phosphatidylcholine. Furthermore PL are required for the formation of chylomicrons, which could be another pathway facilitating the transport of EPA+DHA in the circulating blood. A better understanding of the re-esterification LC PUFA to form TAG and PL, as well as the following integration into lipoproteins requires further investigation.

### Strengths and Limitations

Strengths: Almost identical doses of EPA+DHA were used among treatments and the study had a straightforward design. Recently, we demonstrated substantial inter-individual variability in bioavailability of a TAG form of EPA+DHA in a convenience drink [27]. The effect of inter-individual variability is minimized by our randomized crossover study design, while previous studies largely used randomized parallel designs. Limitations: Despite the study design, we observed high standard deviation values and hence no significant differences in plasma PL FA compositions between groups. Furthermore, the study was a single-dose trial yielding no data on safety and tolerability. Further studies with a larger sample size carried out over a longer period are necessary to substantiate our findings. To match the EPA, DHA and total n-3 FA intake, it was necessary to increase the krill oil capsule intake. Hence the subjects treated with krill oil ingested slightly more fat compared to rTAG and EE (approximately one tenth more in total), which could potentially bias our results. Finally, plasma PL is not representative for tissue, a disadvantage that has been already discussed.

## Conclusion

By comparing plasma PL FA compositions in response to almost identical doses of EPA+DHA in different chemical forms (rTAG vs. EE [both derived from fish oil] vs. krill oil), we demonstrated that EPA+DHA were absorbed in the following order: krill oil > rTAG > EE. While this is the first study to report these differences in bioavailability after oral administration, the study is limited by an endpoint that is not representative for tissue composition. In future long-term studies, such a parameter should be addressed (e.g. the omega-3 index), together with parameters representative for the biological effects of EPA+DHA, such as serum TAG levels, blood pressure and others. Addressing these issues seems important in order to make the use of marine n-3 FA more efficient. Finally, the unexpected high content of free EPA and DHA in krill oil, which might have a significant influence on the bioavailability, should be investigated in more depth and taken into consideration in future trials.

## List of abbreviations

AUC: area under the curve; BMI: body mass index; c_max_: maximum serum concentration; DHA: docosahexaenoic acid (22:6n-3); EPA: eicosapentaenoic acid (20:5n-3); FA: fatty acid; GCP: good clinical practice; LC: long-chain; n-3 FA rTAG: omega-3 fatty acid re-esterified triacylglycerides; n-3 FA EE: omega-3 fatty acid ethyl-ester; PL: phospholipids; rTAG: re-esterified triacylglycerides; t_max_: time to maximum serum concentration.

## Competing interests

C. von Schacky received speaker's honoraria from Solvay/Abbott and grant support from Sanofi-Aventis and Smartfish. He founded Omegametrix, a company offering fatty acid analyses. A. Hahn and J. P. Schuchardt worked as consultants for companies which also produce and merchandise fatty acid supplements. The remaining authors declare no conflicts of interest.

## Authors' contributions

All authors have read and approved the final manuscript. JS was involved in the study design, data analysis, interpretation, and manuscript writing. The study was mainly performed by IS and HM, who were also involved in data analysis and manuscript writing. JN was involved in the preparation of the study. CVS was involved in manuscript writing and editing. The coordinator of the study, AH, was involved in the study design and data interpretation.
